# Endothelial Aryl Hydrocarbon Receptor Nuclear Translocator Mediates the Angiogenic Response to Peripheral Ischemia in Mice With Type 2 Diabetes Mellitus

**DOI:** 10.3389/fcell.2021.691801

**Published:** 2021-06-10

**Authors:** Tu Nguyen, Mei Zheng, Maura Knapp, Nikola Sladojevic, Qin Zhang, Lizhuo Ai, Devin Harrison, Anna Chen, Albert Sitikov, Le Shen, Frank J. Gonzalez, Qiong Zhao, Yun Fang, James J. K. Liao, Rongxue Wu

**Affiliations:** ^1^Biological Sciences Division – Cardiology, Department of Medicine, University of Chicago, Chicago, IL, United States; ^2^Section of Pulmonary and Critical Care, Department of Medicine, University of Chicago, Chicago, IL, United States; ^3^Section of General Surgery, Department of Surgery, University of Chicago, Chicago, IL, United States; ^4^Laboratory of Metabolism, Center for Cancer Research, National Cancer Institute, National Institutes of Health, Bethesda, MD, United States; ^5^Division of Cardiology, Department of Medicine, Inova Heart and Vascular Institute, Annandale, VA, United States

**Keywords:** ARNT, endothelial angiogenesis, hindlimb ischemia, diabetic animal, cell death

## Abstract

Hypoxia-inducible factors (HIFs) are the master regulators of angiogenesis, a process that is impaired in patients with diabetes mellitus (DM). The transcription factor aryl hydrocarbon receptor nuclear translocator (ARNT, also known as HIF1β) has been implicated in the development and progression of diabetes. Angiogenesis is driven primarily by endothelial cells (ECs), but both global and EC-specific loss of ARNT-cause are associated with embryonic lethality. Thus, we conducted experiments in a line of mice carrying an inducible, EC-specific ARNT-knockout mutation (*Arnt*^Δ^ ^EC, ERT2^) to determine whether aberrations in ARNT expression might contribute to the vascular deficiencies associated with diabetes. Mice were first fed with a high-fat diet to induce diabetes. *Arnt*^Δ^ ^EC, ERT2^ mice were then adminstrated with oral tamoxifen to disrupt *Arnt* and peripheral angiogenesis was evaluated by using laser-Doppler perfusion imaging to monitor blood flow after hindlimb ischemia. The *Arnt*^Δ^ ^EC, ERT2^ mice had impaired blood flow recovery under both non-diabetic and diabetic conditions, but the degree of impairment was greater in diabetic animals. In addition, siRNA-mediated knockdown of ARNT activity reduced measurements of tube formation, and cell viability in human umbilical vein endothelial cells (HUVECs) cultured under high-glucose conditions. The *Arnt*^Δ^ ^EC, ERT2^ mutation also reduced measures of cell viability, while increasing the production of reactive oxygen species (ROS) in microvascular endothelial cells (MVECs) isolated from mouse skeletal muscle, and the viability of *Arnt*^Δ^ ^EC, ERT2^ MVECs under high-glucose concentrations increased when the cells were treated with an ROS inhibitor. Collectively, these observations suggest that declines in endothelial ARNT expression contribute to the suppressed angiogenic phenotype in diabetic mice, and that the cytoprotective effect of ARNT expression in ECs is at least partially mediated by declines in ROS production.

## Introduction

Diabetes mellitus (DM) is one of the leading causes of death in the United States and is frequently associated with cardiovascular abnormalities (Hadi and Suwaidi, 2007; Severino et al., 2018; Knapp et al., 2019) that can lead to critical limb ischemia and amputation (Falanga, 2005). Vascular deficiencies are caused by both deterioration of the existing vasculature and impairments in angiogenesis (Costa and Soares, 2013). The angiogenic response to injury and other pathological conditions is chiefly regulated by hypoxia-inducible factors (HIFs) such as HIF1α and aryl hydrocarbon receptor nuclear translocator (ARNT, also known as HIF1β). ARNT is a member of the basic helix-loop-helix/Per-ARNT-SIM (bHLH/PAS) nuclear receptor family (Madan et al., 2002) and regulates the expression of genes involved in cell survival, angiogenesis, and glucose metabolism by dimerizing with HIF1α under hypoxic conditions. Thus, *Arnt* deficiencies in mice lead to angiogenic defects and embryonic lethality, while *Arnt*^–/–^ embryonic stem cells fail to respond to declines in glucose concentration (Maltepe et al., 1997). ARNT also regulates glucose-stimulated insulin secretion in mouse β cells (Pillai et al., 2011) and is severely downregulated (by 98%) in the liver and pancreas of patients with diabetes (Gunton et al., 2005; Pillai et al., 2011), in addition the cardiac-specific loss of ARNT expression in mice leads to a phenotype that mimics diabetic cardiomyopathy (Wu et al., 2014). Hence, ARNT appears to play a key role in diabetes and the pathogenesis of diabetic complications. However, whether changes in ARNT activity may contribute to the vascular deficiencies observed in patients with diabetes remains unclear. Given that endothelial-specific ARNT mutations are generally associated with embryonic lethality (Maltepe et al., 1997), we characterized the angiogenic role of endothelial ARNT expression under diabetic conditions by conducting experiments in mice carrying an inducible, EC-specific ARNT-knockout mutation. The inducible mutation was then induced via oral tamoxifen administration, and the animals were fed with a high-fat diet to induce type 2 DM. Our results indicate that the angiogenic response to peripheral ischemic injury is impaired in diabetic mice and exacerbated by deficiencies in EC ARNT expression. ARNT inactivation is also associated with declines in the angiogenic activity of endothelial cells (ECs) when the cells are cultured under high-glucose conditions.

## Materials and Methods

### Type 2 Diabetes Mellitus Animal Model

Mice with the endothelial-specific knockout of ARNT (*Arnt*^Δ^ ^EC, ERT2^) were generated by crossing *Arnt*^*fl/fl*^ mice with VE-cadherin-CreERT2 mice (Okabe et al., 2014). VE-cadherin-CreERT2 mice were generated by The Jackson Laboratory using sperm from the VE-Cadherin-CreERT2 mouse line, which was a gift from Yoshiaki Kubota (Keio University, Tokyo, Japan) to Liao. ARNT deletion was achieved by oral administration of tamoxifen (30 mg/kg body weight) for 2 weeks, which previously shown to be successful in knocking out ARNT (Wu et al., 2014). The type 2 DM model was induced in *Arnt*^Δ^ ^EC, ERT2^ or control mice (*ARNT*^*fl/fl*^ with tamoxifen administration) using a high-fat diet that contains 62%Kcal of fat, 21%Kcal of carbohydrate, 18%Kcal of protein and 5.21 Kcal/g (A06071302, purchased from Research Diets) for 8 weeks as published previously by others (Winzell and Ahren, 2004; Wang and Liao, 2012).

All high-fat diet fed mice met the criteria for diabetes. db/db mice were purchased from The Jackson Laboratory and studied at 32 weeks of age. These db/db mice were homozygous for the diabetes spontaneous mutation (Lepr^*db*^), which enables the manifestation of a series of conditions: morbid obesity, chronic hyperglycemia, pancreatic beta cell atrophy, and eventual hypoinsulinemia. Their insulin and blood sugar levels start to elevate at 10 to 14 days and at 4 to 8 weeks, respectively. db/+ mice served as the control (Guest and Rahmoune, 2019). The type 1 DM model was induced in 8-week-old male C57BL/6J mice using streptozotocin (STZ) 50 mg/kg body weight. Mice were administered STZ daily at 6 pm via IP injection for five consecutive days. All mice were fasted for 6 h before STZ administration. Mice were used for microvascular endothelial cell isolation 2 weeks after STZ injection. All animal experiments were approved by the Institutional Animal Care and Use Committee (IACUC) at the University of Chicago and followed the National Institutes of Health guide on laboratory animals’ care and use.

### Genotyping

According to the manufacturer’s protocol, genetic DNA was isolated from mouse tails using a DNA isolation kit (QIAGEN DNA isolation kit). Genotyping of Arnt^*fl/fl*^ mice was determined by PCR amplification (forward primer: 5′-CACCTGAGCTAAATTACCAGGCC-3′; reverse primer: 5′-GCATGCTGGCACATGCCTGTCT-3′). Primers were also used for genotyping VE-cadherin-CreERT2 mice (forward primer: 5′-GCG GTC TGG CAG TAA AAA CTA TC-3′; reverse primer: 5′-GTG AAA CAG CAT TGC TGT CAC TT-3′).

### Blood Glucose Measurement and Glucose Tolerance Test

Before blood glucose measurement, mice were fasted for 10–12 h. Approximately 50 μl of blood from lateral tail veins was collected per mouse while animals were awake and restrained. For the glucose tolerance test (GTT), mice were fasted for 6 h. Measurements of blood glucose concentration were performed at 0 min (prior to the glucose injection) and at 15, 30, 60, and 120 min after intraperitoneal glucose injection (2 g glucose/kg body weight). GTT was measured using a Freestyle Precision Neo Blood Glucose Monitoring System (Abbott Diabetes Care Ltd., United Kingdom).

### Hindlimb Ischemia Model and Hemodynamic Measurement

Unilateral hindlimb ischemia was induced and limb perfusion was assessed as previously described (Li et al., 2007). Shortly after, mice were anesthetized by 2% isoflurane. They then underwent operation with ligation and excision of the femoral artery from its origin just above the inguinal ligament to its bifurcation at the root of the saphenous and popliteal arteries. Ligation also included inferior epigastric, lateral circumflex, and superficial epigastric artery branches. We used Doppler perfusion imaging before (0 week) and immediately after surgery at 1–4 weeks.

### Cell Culture

Human umbilical vein endothelial cells (HUVECs) (ATCC, VA, United States) were cultured in M199 growth medium (Invitrogen, CA, United States) supplemented with 20% fetal calf serum (Gibco) and buffered with 25 mM HEPES, fresh L-glutamine (final concentration, 2 mM), 100 U/ml K- penicillin G, and 100 mcg/ml streptomycin sulfate (Biowhittaker). On the day of use, 100 μg/mL heparin (Sigma) and 50 μg/mL Endothelial Cell Growth Supplement (ECGS, Biomedical Technologies, Inc.) were added to get a final concentration of 0.1 mg/ml of medium. Cells were cultured in T25 vented flasks with a humidified atmosphere of 95% air and 5% CO_2_ at 37°C.

### Isolation of Murine Skeletal MUSCLE MICROVASCULAR ENDOTHELIAL CELLS (MMVECs)

This method was partly adapted from various studies and protocols focused on isolating ECs from mice (Nakano et al., 2018; Wang et al., 2019), which used mechanical and enzymatic dissociation followed by activated magnetic cell sorting for purification. In brief, the musculus quadriceps femoris was harvested from mice by cutting the tendon from the knee and severing the muscle from the femur to the hip. About 1 g of muscle tissue was finely minced in a solution containing 2 mg/ml collagenase (S5B115583 from Worthington) and PBS with calcium and magnesium (14040-133 from GIBCO) and incubated on a shaker at 37°C for 1 h. After the incubation period, the supernatant was harvested and centrifuged to collect the pellet. Then, the pellet was re-suspended in a solution containing PBS without calcium and magnesium (GIBCO), BSA (Bovine Serum Albumins) (0.1% v/v, Sigma), and platelet endothelial cell adhesion molecule-1 (PECAM1, CD31) (Choi et al., 2015) coated beads (BDB553370, Fisher). After that, the mixture was rotated for 10 min at room temperature and mounted on a magnetic separator. Following 10–12 washes, the cells were re-suspended in growth media containing DMEM with 1 g/L glucose (11885-084 from GIBCO), 20% FBS (v/v from GIBCO), 1% penicillin/streptomycin, 150 μg/mL Endothelial Cell Growth Supplement (ECGS, Biomedical Technologies), and 100 μg/mL heparin (Sigma). The cells were incubated in a 5% CO2 (v/v) and 95% (v/v) air incubator. After the cells reached 90% confluence, we harvested the cells and re-suspended them in PBS without Calcium and Magnesium and BSA (0.1% v/v). Intercellular Adhesion Molecule 2 antibody was used for the second sort.

### Isolation and Measurement of ARNT mRNA and Protein

Total RNA was isolated from microvascular endothelial cells (MVECs) derived from mouse organs using Direct-zol RNA Mini-Prep kit (Zymo Research, CA, United States) according to the manufacturer’s protocol. cDNA was synthesized from 500 ng of RNA using iScript Reverse Transcription Supermix for RT-qPCR (Bio-Rad Laboratories Inc., CA, United States). Quantitative real-time PCR was performed using iTaq Universal SYBR Green Supermix (Bio-Rad Laboratories Inc., CA, United States) in CFX Connect Real-time System (Bio-Rad Laboratories Inc., CA, United States). qPCR results were normalized to the expression of the endogenous control 18S. Fold changes in the transcripts were determined using the delta cycle threshold (i.e., ΔΔCt) method. The forward and reverse primer sequences used for human ARNT were: 5′-GAA CCA GCC ACA GTG TGA ATG G-3′ and 5′-GAG CCC ATA CAC ATC CTC ATG GAA GAC TGC TG-3′, respectively. All samples were analyzed in triplicates. 18S mRNA levels were used as an internal quantitative control. Endothelial ARNT protein levels from MVECs isolated from DB/DB and control mice were determined by Western blot, as we have published before (Wu et al., 2014). Antibody for ARNT was purchased from Cell Signaling (Cat# 5537). Antibody for Cofilin served as an internal loading control (Abcam, Ca# 42824).

### Cell Transfection and Treatment

ARNT knockdown was induced by small interfering RNA (siRNA) directed against ARNT (Ambion, Carlsbad, CA, United States) in HUVECs. HUVECs were transfected with 25 nM of non-targeted or ARNT siRNA mixed with the siRNA transfection reagent Dharma FECT 3 (GE Healthcare Dharmacon). The mixtures were incubated in serum-free medium for 20 min at room temperature before being added to the HUVECs. siRNA transfection was allowed to proceed for 48 h before harvesting whole cells for RNA isolation for real-time PCR. Cells were treated with high glucose followed by 12 h of starvation. Cells in the high glucose (HG) group were cultured in 35 mmol/L D-glucose media (Sigma). Identical concentrations of medium containing 5.5 mmol/L D-glucose plus 29.5 mmol/L mannitol served as the normal glucose (NG) control group. To test whether reactive oxygen species (ROS) inhibitors have a role in the rescue of cell death, MMVECs were pretreated with PBS or mito-TEMPO (Sigma-Aldrich) at 0.5 μM for 30 min before HG was added.

### Cell Proliferation and Cell Apoptosis Assay

After transfection with either ARNT-siRNA or control-siRNA, HUVECs (1.1 × 10^5^) were incubated in FBS-free medium for 6 h and then in HG or NG culture medium. HUVECs were harvested from wells using 0.25% trypsin and counted every 24 h for 3 days using the Moxi GO II (Offlo Technologies) according to the manufacturer’s protocol. Cell survival in HUVECs and MMVECs was determined by a cell apoptosis assay following 48 h of HG or NG treatment. The cells were washed with PBS and double-stained with FITC-conjugated Annexin V and PI using the Annexin V and PI kit (Offlo Technologies) according to the manufacturer’s protocol. Measurements were obtained via Moxi GO II (Offlo Technologies).

### *In vitro* Cell Migration Assessment

Muscle microvascular endothelial cells isolated from mouse skeletal muscle were seeded at a density of 2.0 × 10^4^ cells in Incucyte ImageLock 96 well microplate Essen ImageLock^TM^ overnight with HG or NG media to let cells attach to the well. We then used a WoundMaker^TM^ device (4563, Essen Bioscience) containing 96 pins to scratch homogeneous micron-wide wounds through all the cell monolayers. After washing with PBS, 100 μl of fresh serum-free medium with either NG or HG were added to each well. The plates were then placed into the Incucyte S3 instrument (Essen Bioscience) to scan for relative wound width and confluence every 2 h for 48 h total. Data were analyzed through the instrument’s software in accordance with the manufacturer’s analysis manual.

### Tube Formation Assay

Passage 2–3 HUVECs (followed by ARNT siRNA transfection for 48 h) were cultured at a density of 1.2 × 10^5^ with HG or NG medium in 24 well plates coated with 300 μl of Growth Reduced Matrigel (Corning, NY, United States). After 17 h, the formation of tube-like structures was visualized under a microscope. Images were taken, and the number of meshes, number of extremities, length of branches and segment length were measured and analyzed by the angiogenesis analyzer for Image J, as previously published (Severino et al., 2018).

### Measurement of ROS Production

Cultured MMVECs from either ecARNT-/- or control mice were seeded at a density of 2.0 × 10^4^ cells in 96 well plates. Following incubation with NG or HG media for 48 h, the ROS levels were assessed using the Cellular ROS/Superoxide Detection Assay Kit (ab139476 from Abcam), which has been widely and successfully utilized in many different studies (Hu et al., 2019, 2020; Ren et al., 2019; You et al., 2020). The cells were prepared for measurement as instructed by the manufacturer’s manual and submitted to the Biotek Synergy two microplate reader set with standard fluorescein (Ex = 488 nm, Em = 520 nm) and rhodamine (Ex = 550 nm, Em = 610 nm).

### Statistical Analysis

Results were compared using one-way or two-way analysis of variance (ANOVA) as appropriate followed by the Tukey–Kramer *post hoc* test using GraphPad Prism statistics software (GraphPad Software, Inc.). Data are shown as means ± SEM. *P*-values < 0.05 were considered to be significantly different.

## Results

### Endothelial ARNT Expression Is Reduced in Diabetic Mice

**FIGURE 1:**
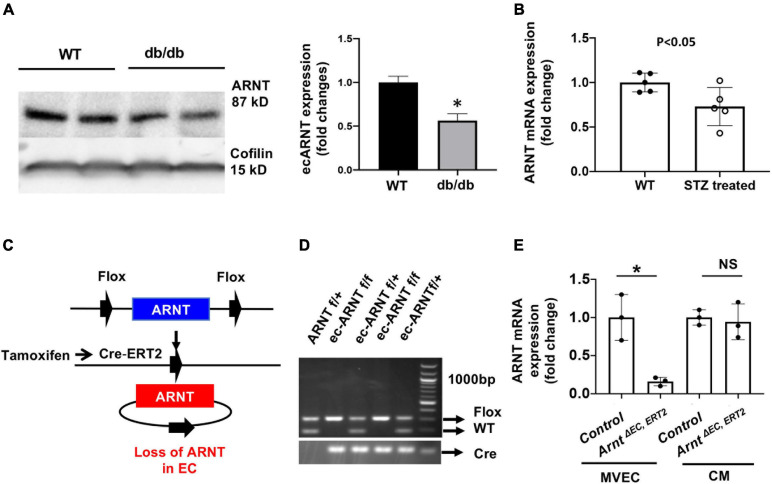
Endothelial ARNT expression in diabetic animals and the generation of inducible endothelial-specific ARNT-deficient adult (*Arnt*^Δ^ ^EC, ERT2^) mice. **(A)** A Representative Western blot and comparison graph of ARNT protein expression levels in mouse microvascular endothelial cells (MVECs) isolated from control (db/+) and diabetic mouse (db/db) organs. **(B)** *ARNT* mRNA expression levels in MVECs isolated from control and STZ-treated diabetic mouse organs. **(C)** Schematic diagram of ecARNT deletion. **(D)** PCR analysis of genomic ARNT DNA. **(E)** *ARNT* mRNA expression levels in MVECs and isolated cardiomyocytes (CM). Data are presented as the mean ± SEM (*n* = 3–6) mice per group. ^∗^*P* < 0.01 versus WT or control mice.

We began investigating the role of endothelial ARNT in diabetes by measuring ARNT expression in MVECs isolated from the hearts of diabetic mice. In models of type 1 [streptozotocin [STZ]-induced (Graham et al., 2011)] and type 2 (db/db) diabetes, the abundance of ARNT protein (DB/DB, Figure 1A) or mRNA (STZ-induced, Figure 1B) was significantly lower in MVECs from diabetic mice than in those from non-diabetic mice. *In vitro*, we were not able to detect a significant reduction in ARNT mRNA in response to HG; however, cell culture with prolonged HG exposure caused a decrease of *Arnt* mRNA in HUVECs (Supplementary Figure 1). To explore the role of ecARNT in angiogenesis during diabetes, we conducted a series of studies in mice carrying a tamoxifen-inducible, EC-specific ARNT-knockout (*Arnt*^Δ^ ^EC, ERT2^) mutation. The mutant line was generated by breeding Arnt^flox/flox^ mice in which exon 6 of Arnt is flanked by loxP sequences (Wu et al., 2014), with VE-cadherin-Cre^*ERT*2^ mice expression of Cre-recombinase is tamoxifen-inducible and regulated by the vascular endothelial (VE) Cadherin promoter (Han et al., 2014; Okabe et al., 2014; Figure 1C). After cross-breeding for two generations, PCR analyses of genomic DNA confirmed that the *Arnt*^Δ^ ^EC, ERT2^ genotype was present in ∼1/4 of offspring (Figure 1D), and in the absence of tamoxifen treatment, the mice were phenotypically indistinguishable from wild-type mice. Because intraperitoneal injections of tamoxifen have been associated with Cre-driven cardiomyopathy in similar mouse models (Lexow et al., 2013), the *Arnt*^Δ^ ^EC, ERT2^ mice were orally treated with tamoxifen for 2 weeks via our published protocols (Ichikawa et al., 2014; Wu et al., 2014). Cell-type specificity of the *Arnt* disruption in *Arnt*^Δ^ ^EC, ERT2^ mice was confirmed by comparing *ARNT* mRNA abundance in ECs and other cells from *Arnt*^Δ^ ^EC, ERT2^ mice that had not been treated with tamoxifen (Figure 1E).

### EC-Specific Loss of ARNT Expression Impairs the Angiogenic Response to Peripheral Ischemic Injury in Mice With Type 2 DM

**FIGURE 2:**
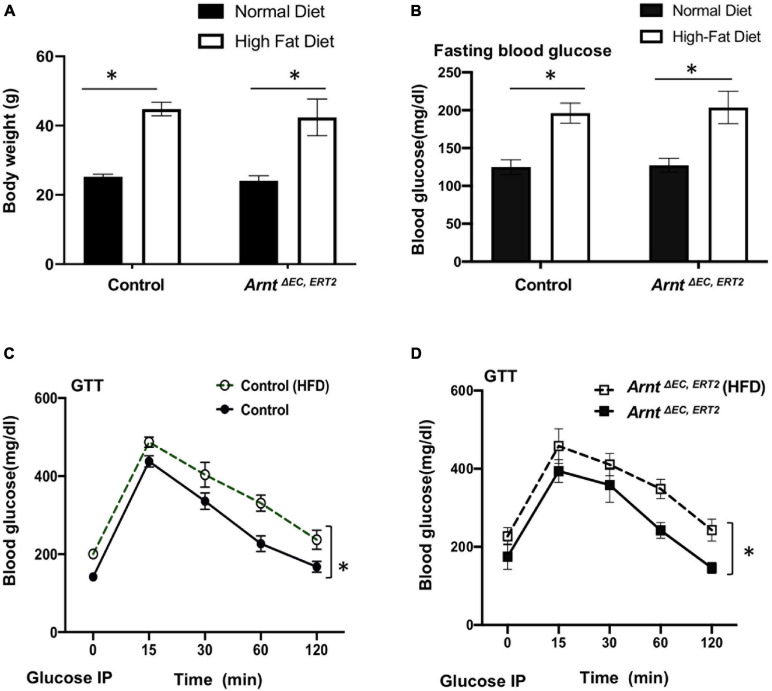
High-fat diet (HFD) induced type-2 diabetic model in both WT control and *Arnt*^Δ^ ^EC, ERT2^ mice. **(A)** Bodyweight in control and *Arnt*^Δ^ ^EC, ERT2^ mice placed on both a standard diet and HFD for 8 weeks as indicated. **(B)** Fasting plasma glucose level in control and *Arnt*^Δ^ ^EC, ERT2^ mice after 8 weeks of standard diet and HFD for as indicated. **(C,D)** Glucose tolerance test (GTT) of control and *Arnt*^Δ^ ^EC, ERT2^ mice after 8 weeks of standard diet and HFD for as indicated. Glucose was administered as a 0.2 g/ml/100 g IP injection. Data are presented as the mean ± SEM (*n* = 5–8) mice per group. ^∗^*P* < 0.05, ^∗∗^*P* < 0.01 versus control mice.

**FIGURE 3:**
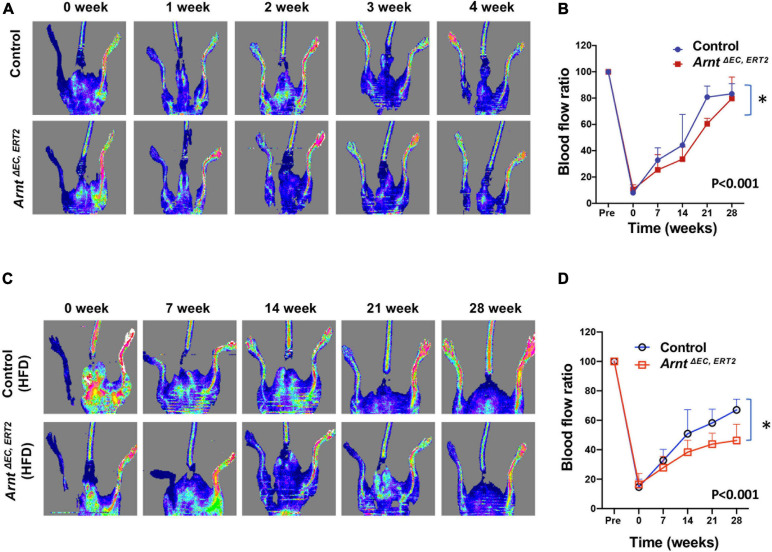
Laser Doppler blood flow analysis for the ischemic hindlimb of *Arnt*^Δ^ ^EC, ERT2^ or wild-type control mice with and without high-fat diet (HFD). **(A)** Laser Doppler perfusion imaging of blood flow in the ischemic hindlimb measured in control and *Arnt*^Δ^ ^EC, ERT2^ mice fed with a standard diet. Laser Doppler perfusion imaging of blood flow in the ischemic hindlimb was measured immediately (time 0) and 1–4 weeks after surgery. Blood flow is color-coded, with normal perfusion indicated by red and a marked reduction in blood flow indicated by blue. **(B)** Quantitation of blood flow is expressed as the ratio of blood flow in the ischemic (left) hindlimb to that in the normal (right) hindlimb. **(C)** Laser Doppler perfusion imaging of blood flow in the ischemic hindlimb measured in control and *Arnt*^Δ^ ^EC, ERT2^ diabetic mice induced with 8 weeks of HFD. **(D)** Quantitation of blood flow as in B. Data are means of values from 8–11 animals per group. ^∗^*P* < 0.01 versus corresponding value for wild-type mice.

When fed with a high-fat diet, both control and *Arnt*^Δ^ ^EC, ERT2^ mice developed diabetes, as evidenced by increases in body weight (Figure 2A) and in blood-glucose levels both under fasting conditions (Figure 2B) and in response to intraperitoneal glucose injection (i.e., the GTT, Figures 2C,D). Notably, measurements of glucose tolerance did not differ significantly between *Arnt*^Δ^ ^EC, ERT2^ and control mice under either normal or high-fat feeding conditions (Supplementary Figure 2), which suggests that ecARNT expression might not influence the development of type 2 diabetes. We next investigated whether ecARNT expression could contribute to ischemia-induced angiogenesis under diabetic conditions. We utilized the murine model of hindlimb ischemia (Niiyama et al., 2009) and evaluated perfusion in the limbs of mice before, immediately after, and 7–28 days after surgically induced hindlimb ischemia (HLI). Blood flow was quantified via laser doppler perfusion imaging, and measurements in the injured limb were normalized to measurements in the uninjured contralateral limb. In non-diabetic animals (i.e., under normal dietary conditions), perfusion of the injured limb was lower in *Arnt*^Δ^ ^EC, ERT2^ mice compared to the control mice on day 7 (1 week), 14 (2 weeks), and 21 (3 weeks) after HLI induction (Figures 3A,B). However, measurements in the two groups were similar on day 28 (4 weeks) (Figures 3A,B). In diabetic mice (i.e., those fed a high-fat diet), recovery was impaired in both control and ecARNT-KO groups (Supplementary Figure 3), which is consistent with the observations previously reported (Hazarika et al., 2007). Also, the difference between measurements in *Arnt*^Δ^ ^EC, ERT2^ and control mice became progressively greater through day 28 (Supplementary Figure 4 and Figures 3C,D). Hence, the loss of ecARNT expression evidently appears to have exacerbated the diabetes-induced impairment of ischemia-induced angiogenesis.

### ARNT Inactivation Impairs the Angiogenic Activity of ECs in High-Glucose Conditions

**FIGURE 4:**
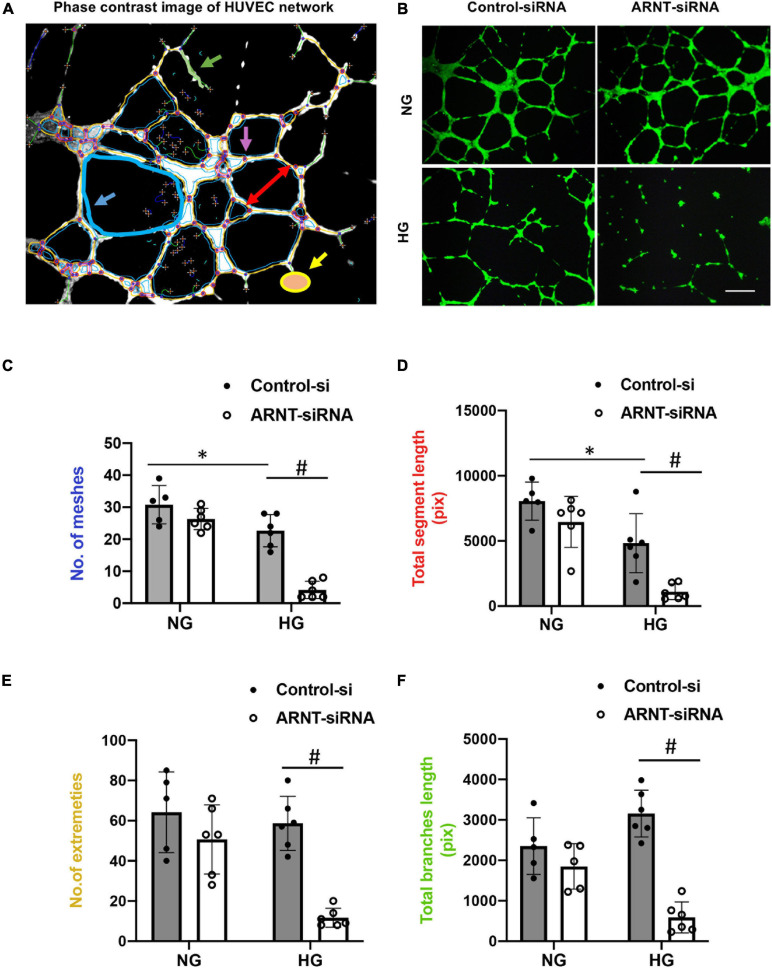
The effects of ARNT knockdown on tube formation in cultured human umbilical vein endothelial cells (HUVECs). Cells were plated on Matrigel pre-coated 24-well plates that were transfected with either ARNT-siRNA or control-siRNA followed by high glucose (HG) and control exposure (NG, identical concentrations of mannitol as an osmotic control group) for an additional 48 h. **(A)** Representative image of HUVEC network, analyzed by the angiogenesis analyzer for Image J. The picture shows meshes (blue), extremities (yellow), branches (green), and segment length (red) as indicated. **(B)** Images of capillary-like tube formation, which mature by 6–16 h. Photomicrograph shows the effect of 35 mM high glucose exposure on tube formation with and without ecARNT knockdown (Scale bar = 200 μm). Summary of analysis is shown. **(C)** Number of meshes. **(D)** Total segment length. **(E)** Number of extremities. **(F)** Total branch length. Data are presented as the mean ± SEM (*n* = 5–6). ^∗^*P* < 0.01 versus Control-si (NG), ^#^*P* < 0.01 vs. Control-si (HG).

To characterize how deficiencies in endothelial ARNT expression alter the activity of ECs in diabetic animals, HUVECs were transfected with a vector coding for either ARNT-siRNA or a control-siRNA. ARNT knockdown was confirmed by both mRNA and protein expression (Supplementary Figure 5). Cells were then cultured under normal or high (35 mM) glucose (NG or HG, respectively) conditions. The NG condition was cultured in medium containing 5.5 mmol/L D-glucose supplemented with 29.5 mM mannitol to ensure osmotic equivalence. When HG-cultured cells were plated onto Matrigel, measurements of tube formation were significantly lower in ARNT-siRNA–transfected HUVECs compared to control-transfected HUVECs, but measurements in the two groups of NG-cultured cells were similar (Figures 4B–F). The impaired tube formation was determined by the number of meshes (Figure 4C), segment length (Figure 4D), number of extremities (Figure 4E), and length of branches (Figure 4F), which was analyzed by the angiogenesis analyzer for Image J. Of note, to assist our analyses, we used phase contrast of HUVEC network as indicated (Figure 4A).

**FIGURE 5:**
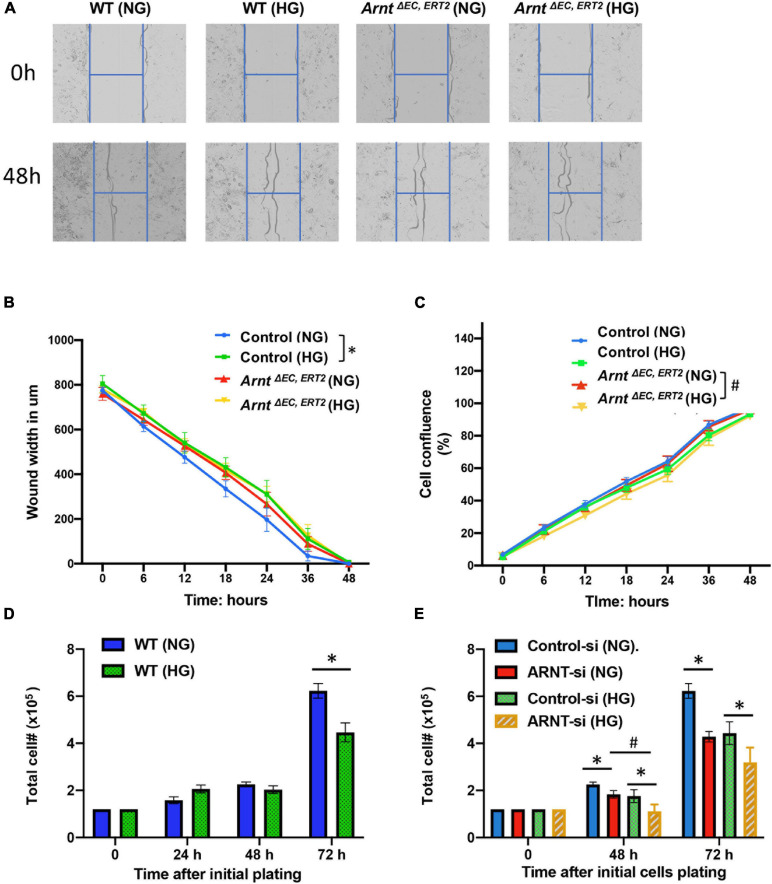
Effect of ecARNT knockdown on high glucose-induced migration dysfunction in MMVEC and proliferation inhibition in HUVECs. **(A)** Representative micrographs of automatically acquired images of real-time live cells using Incucyte^®^ system. Images show that compared to WT HUVECs in osmotic pressure control group (WT and NG), HUVECs with high glucose migrated more slowly *P* = 0.0026, corresponding statistical analysis of HUVEC migration by scratch wound healing assay. All 96-well kinetic trends were viewed at once with Incucyte^®^ PlateGraph and data was exported to calculate different values shown. **(B)** Wound width. *n* = 8, *p < 0.01 vs. WT (NG). Please note, migration in HUVECs with ARNT knockdown failed to respond to high glucose exposure **(C)** Cell confluence%, *n* = 8, *p < 0.001 vs. WH (NG); ^#^*P* < 0.01 vs. *Arnt*^Δ^ ^EC, ERT2^ (NG). **(D)** High glucose-mediated reduction in WT HUVEC proliferation over time. **(E)** Effect of ecARNT knockdown on HUVEC proliferation over time following either normal glucose or high glucose exposure. The same number of HUVECs were seeded on Day 0 (1.2 × 10^5^) in 6- well plate. ARNT knockdown was induced with an siRNA transfection. Data are presented as the mean ± SEM (*n* = 6). ^∗^*P* < 0.01 versus corresponding control.

Cell migration and proliferation toward angiogenic stimuli are essential factors for tube formation. Therefore, we performed a cell wound healing assay using an advanced live-cell imaging and analysis system (Incucyte^®^ S3) (Figure 5A). The data suggested that *in vivo* wound closure was impaired by high glucose in cells, but not by the loss of ARNT, as migration in MMVECs from *Arnt*^Δ^ ^EC, ERT2^ mice failed to respond to HG (Figure 5B). However, cell confluence, used as an indicator of cell proliferation, demonstrated a reduction in MMVECs from ecARNT KO mice when cultured in HG conditions for 2 days (Figure 5C). We also measured cell proliferation in HUVECs in the presence of HG or NG conditions for 24 to 72 h. Inconsistent with the previous observation, the results showed that cell proliferation for WT HUVECs was inhibited when treated with high glucose for 72 h, and this inhibition was not due to osmotic pressure (Figure 5D). ARNT knockdown-mediated inhibition of cell proliferation was greater than control HUVECs in the presence of HG (Figure 5E). Thus, knockdown of ecARNT expression inhibits cell proliferation, which might have contributed to impaired tube formation in HG conditions, suggesting an essential role for ARNT in angiogenesis in diabetic mellitus. This is in line with our *in vivo* studies.

**FIGURE 6:**
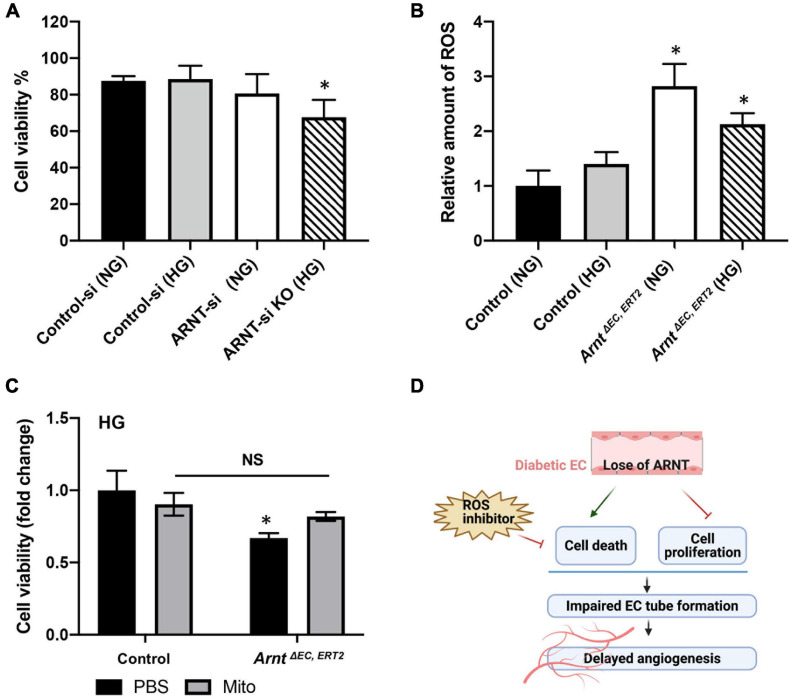
Effects of ARNT expression on cell viability and cellular reactive oxygen species (ROS) production under either high glucose (HG) or normal media (NG) conditions. **(A)** Cell viability in the presence of high glucose or control media with and without ARNT deletion in HUVECs as indicated. ARNT knockdown was induced with an siRNA transfection. The treatment of a control siRNA serves as control. **(B)** ROS levels in MMVECs isolated from *Arnt*^Δ^ ^EC, ERT2^ and *Arnt*^*fl/fl*^ WT mice under high glucose. The ROS levels were assessed using the Cellular ROS/Superoxide Detection Assay Kit from Abcam. **(C)** Effect of inhibition of ROS by Mito-TEMPO on cell viability. Isolated MMVECs from *Arnt*^Δ^ ^EC, ERT2^ and *Arnt*^*fl/fl*^ WT control mice were cultured for 48 h in HG media followed by 24 h of administration of Mito-TEMPO at 0.5 μM. Cell viability was measured as before using the Moxi GO II machine. The data are shown as mean ± SEM (*n* = 6–8 per group). ^∗^*P* < 0.01. **(D)** Schematic diagram of the role of endothelial ARNT in diabetic angiogenesis.

Aryl hydrocarbon receptor nuclear translocator inactivation was also associated with declines in HUVEC viability when the cells were cultured under HG conditions (Figure 6A), and the production of ROS was significantly greater in skeletal-muscle MVECs from *Arnt*^Δ^ ^EC, ERT2^ mice compared to control mice under both NG and HG conditions (Figure 6B). Notably, measures of cell viability under HG conditions were also significantly lower for *Arnt*^Δ^ ^EC, ERT2^ MVECs than for control MVECs (Figure 6C). In addition, cell viability increased in *Arnt*^Δ^ ^EC, ERT2^ MVECs when the cells were treated with an ROS inhibitor (Mito-TEMPO, 0.5 μM) (Figure 6C; Dikalova et al., 2010; Vaka et al., 2018), which suggests that the cytoprotective effect of ARNT in ECs is mediated by declines in ROS production.

## Discussion

We are the first to report a reduction in ARNT in mouse MVECs isolated from diabetic animals. We successfully generated mice with an inducible endothelial-specific deletion in *A*rnt (ecARNT KO mice) and showed that these mice have a delayed angiogenic response to peripheral ischemic injury. In addition, loss of ARNT deletion in ECs exacerbated the diabetes-mediated impairment of ischemia-induced angiogenesis. Our *in vitro* studies found that genetic disruption or knockdown of ARNT worsened high glucose-induced endothelial cell dysfunction as measured by the angiogenic response in HUVECs (i.e., cell proliferation and tube formation). Interestingly, ARNT-induced cell death and reduced viability were associated with HG, but not NG cultured cells.

Moreover, we demonstrated that ROS production was significantly elevated in ecARNT KO MMVECs. Under high glucose conditions, increased cell death in ARNT KO MMVECs was rescued using Mito-Tempo (a mitochondrial ROS inhibitor), indicating ARNT protects cells from damages caused by ROS generation during high glucose conditions. Thus, our study demonstrates a direct role for ecARNT in angiogenesis and as an antioxidant in the setting of diabetic complications. Although many factors are likely contributing to impaired angiogenesis in diabetes, our study is the first to report alterations of ARNT in the microvascular system as a major contributor to the antioxidant process.

The HIF1//ARNT complex is activated in response to hypoxia. Diabetic tissue damage leads to hypoxia. However, HIF-1 signaling is impaired in diabetes, which may contribute to the development of diabetic complications (Catrina and Zheng, 2021). ARNT has been implicated in the development and progression of diabetes and is severely downregulated in the liver and pancreas of patients with diabetes (Gunton et al., 2005; Pillai et al., 2011). In this study, we observed a reduction in ARNT expression in MVECs isolated from both types 1 and 2 diabetic animal models, which may be caused by high glucose. Additionally, we focused on the role of ARNT in MVECs under hyperglycemic conditions in mice with diabetes. ARNT has been widely studied for its crucial role in angiogenesis. Overexpression of ARNT has previously been shown to rescue growth-restricted fetoplacental angiogenesis (Ji et al., 2019). In addition, ARNT may play a role in tumor angiogenesis via its capacity to bind xenobiotic responsive elements to induce transcription (Hankinson, 1995; Maltepe et al., 2000; Ji et al., 2019). Despite the wide array of studies of ARNT in angiogenesis, the understanding of ARNT’s roles in angiogenic abnormalities in DM has been limited so far. In our study, in the absence of ischemia, *Arnt*^Δ^ ^EC, ERT2^ mice did not display any vascular abnormalities at baseline, but hindlimb ischemia-induced angiogenic response was slightly delayed in *Arnt*^Δ^ ^EC, ERT2^ mice compared to control mice. This impairment was much more pronounced under diabetic conditions. Of note, some diabetic mice with *Arnt*^Δ^ ^EC, ERT2^ even developed leg necrosis post hindlimb ischemia.

Given that ARNT is a master regulator of angiogenesis (Su et al., 2015; Ji et al., 2019), its diminished expression in the endothelium in the setting of diabetes may explain why diabetes is the biggest risk factor leading to vascular endothelial dysfunction and delayed tissue repair and regeneration, which eventually manifests as severe vascular complications in diabetic patients (Hadi and Suwaidi, 2007; Severino et al., 2018; Knapp et al., 2019). ARNT reduction in MVECs was observed in both types 1 and 2 diabetic mouse models, suggesting that hyperglycemia, rather than insulin resistance, impairs endothelial ARNT activity. Hence, we used high glucose media to treat HUVECs or primary MMVECs in order to examine angiogenic activity. To be diagnosed with diabetes, one of the criteria that must be met is a blood glucose level greater than or equal to 30 mM (200 mg/dl). Therefore, we used a culture medium containing 35 mmol glucose as our high-glucose (HG) condition and 5.5 mM (100 vmg/dl) glucose plus 29.5 mmol/L mannitol (as an osmotic control) for our normal glucose (NG) control group. Our data indicated that compared with the control group, ARNT knockdown (KD) using siRNA in HUVECs significantly worsened the HG induced impairment in tube formation, which is in line with our *in vivo* observations. Our results are in agreement with previous studies of wounds from diabetic patients, which showed decreased cellular migration, proliferation, and cell survival under HG conditions (Lan et al., 2009). However, ARNT KD did not affect cell migration under HG conditions. HG-mediated inhibition of proliferation in HUVECs was worsened with ARNT KD and HG-induced cell death was only observed in ARNT KD HUVECs, not in control HUVECs. This suggests an important role for ARNT in regulating EC proliferation and cell death under HG conditions, a process that is important in tube formation and angiogenesis.

High glucose in diabetes increases the production of ROS, which can impair cellular functions such as migration and proliferation, and activate apoptosis in β-cells (Volpe et al., 2018). Our results showed that the ROS levels were notably elevated in ARNT-KO MMVECs as compared to control MMVECs both in NG and HG conditions. We then conducted an experiment that inhibited ROS production using Mito-TEMPO (Dikalova et al., 2010; Vaka et al., 2018) and observed a rescuing effect on cell death in the *Arnt*^Δ^ ^EC, ERT2^ HG group. Collectively, these findings suggest an important role for ARNT in acting as an antioxidant responder to protect MVECs under high glucose conditions. This conclusion corroborates a similar finding of the antioxidant role of ARNT previously observed in leukemia (Gu et al., 2013). Although our study demonstrated the critical antioxidant action of ARNT against ROS, it did not reveal the causative relationship between ARNT and the production of ROS. Thus far, knocking down ARNT has been shown to suppress the expression of PDK1 (pyruvate dehydrogenase kinase (1) in certain cancers. This in turn activates PDH (pyruvate dehydrogenase), the TCA cycle enzyme that converts pyruvate to acetyl Co-A to fuel the TCA cycle for oxidative phosphorylation (OXPHOS) (Kim et al., 2006; Huang et al., 2021). The upregulation of OXPHOS in mitochondria ultimately leads to increased ROS production (Li et al., 2013; Reichart et al., 2019; Huang et al., 2021). Unquestionably, these findings are valuable in supporting the causative relationship between ARNT and ROS production but limit ARNT’s actions in ROS production to the PDK1-dependent pathway in cancer cells. Nevertheless, the underlying mechanisms in which ARNT could be involved in ROS generation in mitochondria under diabetic conditions is not clear. It is known that PDK4 levels are elevated in diabetes (Wu et al., 1998, 2000; Sugden and Holness, 2002). Therefore, it is in our best interest into investigating whether ecARNT in diabetes would regulate the production of ROS resulting from the upregulation of OXPHOS via a novel PDK4 dependent pathway or through the same pathway regulated by PDK1 above. This future direction is valuable because it will not only further elucidate our understanding of diminished cell proliferation and impaired angiogenesis via a ROS perspective but also allow us to expand our knowledge of the importance of ARNT in regulating genes encoding for glycolytic enzymes to produce energy, which many other studies have proved (Firth et al., 1994, 1995; Semenza et al., 1994, 1996). Despite these promising future directions, this study is the first to establish the connection between ecARNT and impaired angiogenesis in diabetes and demonstrate that ecARNT deficiency in diabetes mellitus exacerbates angiogenesis impairment via a mechanism that intensifies the negative effects of ROS.

## Data Availability Statement

The raw data supporting the conclusions of this article will be made available by the authors, without undue reservation.

## Ethics Statement

The animal study was reviewed and approved by the UCMC Institutional Review Boards at the University of Chicago.

## Author Contributions

TN and RW conceived of the study design and wrote the manuscript. TN, MZ, MK, and NS performed experiments with assistance from QINZ, LA, DH, AC, AS, and LS. TN, MZ, MK, and NS analyzed the data. FG, YF, QIOZ, and JL reviewed and contributed to the writing of the manuscript. All authors read and approved the final version of the manuscript.

## Conflict of Interest

The authors declare that the research was conducted in the absence of any commercial or financial relationships that could be construed as a potential conflict of interest.
