# Supplementary material for: Endothelial Aryl Hydrocarbon Receptor Nuclear Translocator Mediates the Angiogenic Response to Peripheral Ischemia in Mice With Type 2 Diabetes Mellitus
Source: Front Cell Dev Biol. 2021 Jun 10;9:691801. doi: 10.3389/fcell.2021.691801 (PMC8222825; doi:10.3389/fcell.2021.691801)
Supplement: Supplementary file 1 [file Data_Sheet_1.PDF]

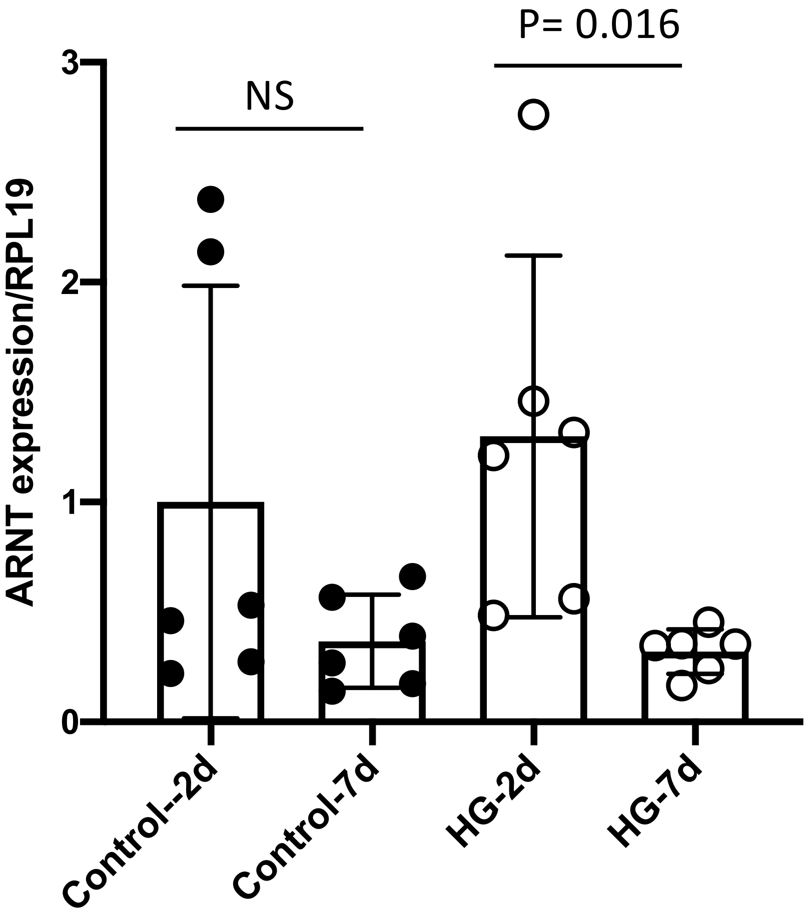

Supplemental figure 1. ARNT mRNA expression in response to HG treatment by real time PCR (RT-PCR). HUVECs were cultured in 35 mmol/L D-glucose media. Identical concentrations of medium containing 5.5 mmol/L D-glucose plus 29.5 mmol/L mannitol served as the normal glucose control group. Ribosomal Protein L19 (RPL19) was used as in internal control. Data are presented as the mean ± SEM (n= 6, P<0.016 versus corresponding 2d cell culture).

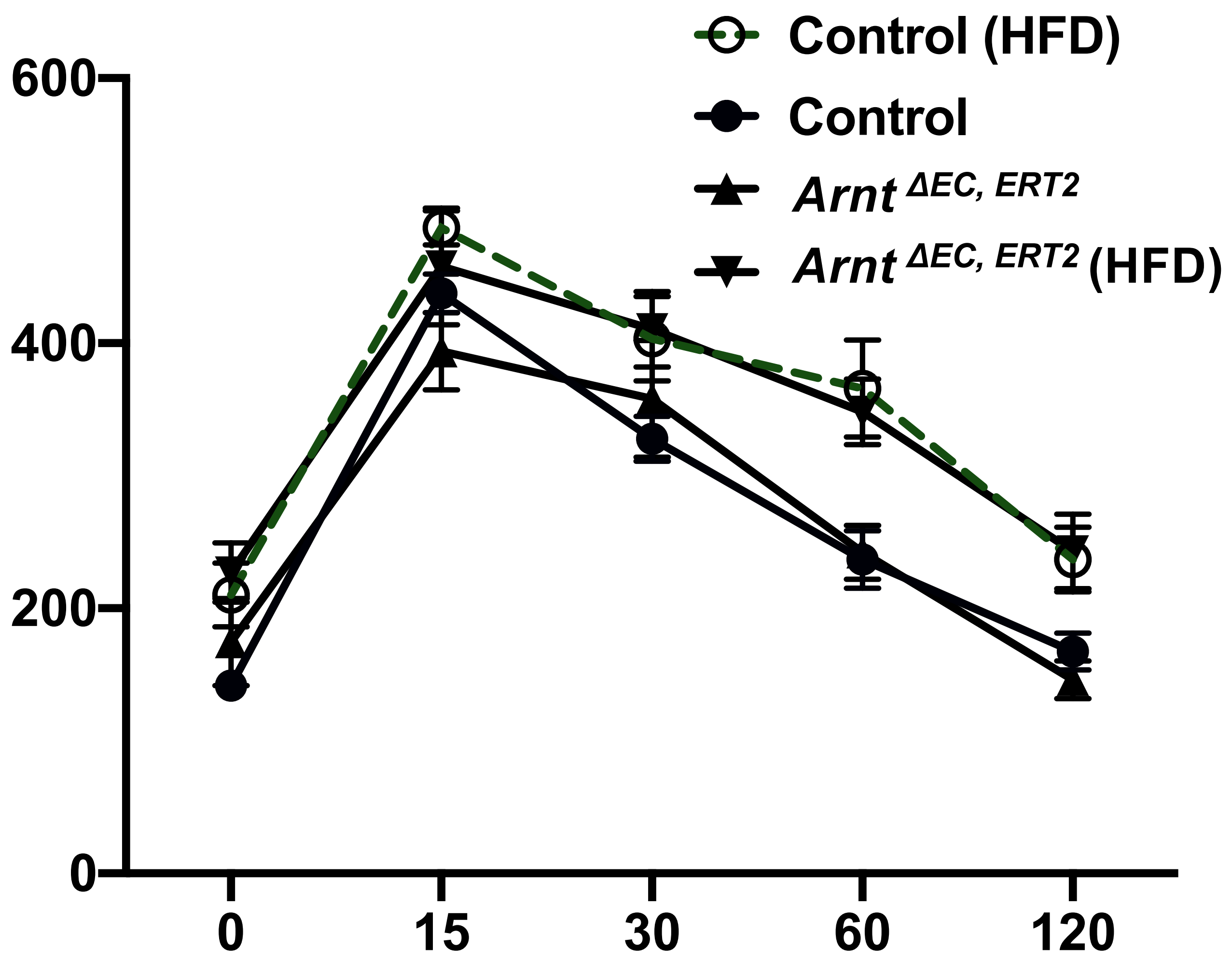

Supplemental figure 2. Glucose tolerance test (GTT) in high-fat diet (HFD) induced type-2 diabetic model in both WT control and *Arnt* $^{\Delta EC,\ ERT2}$ mouse. Data are presented as the mean ± SEM (n= 5-8 mice).

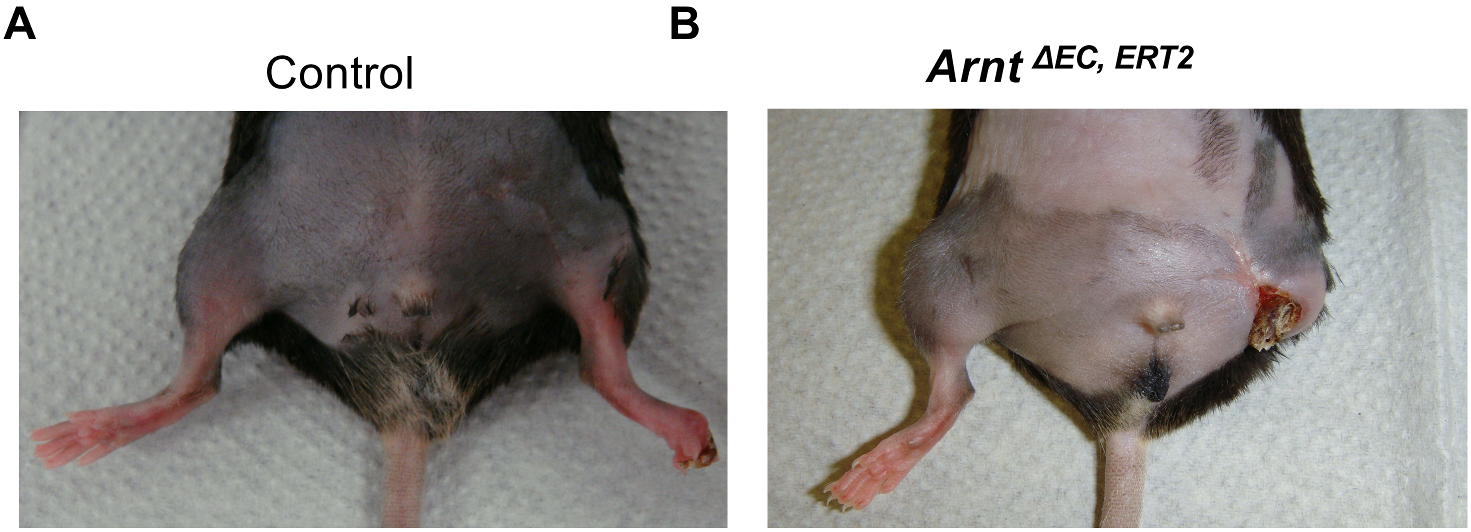

Supplemental figure 3. Representative image of diabetic mice 2 weeks after ischemic hindlimb operation. Diabetic mice were induced with high-fat diet (HFD) administration for 12 weeks. A) Control mouse. B) *Arnt* $^{\Delta EC,\ ERT2}$ mouse

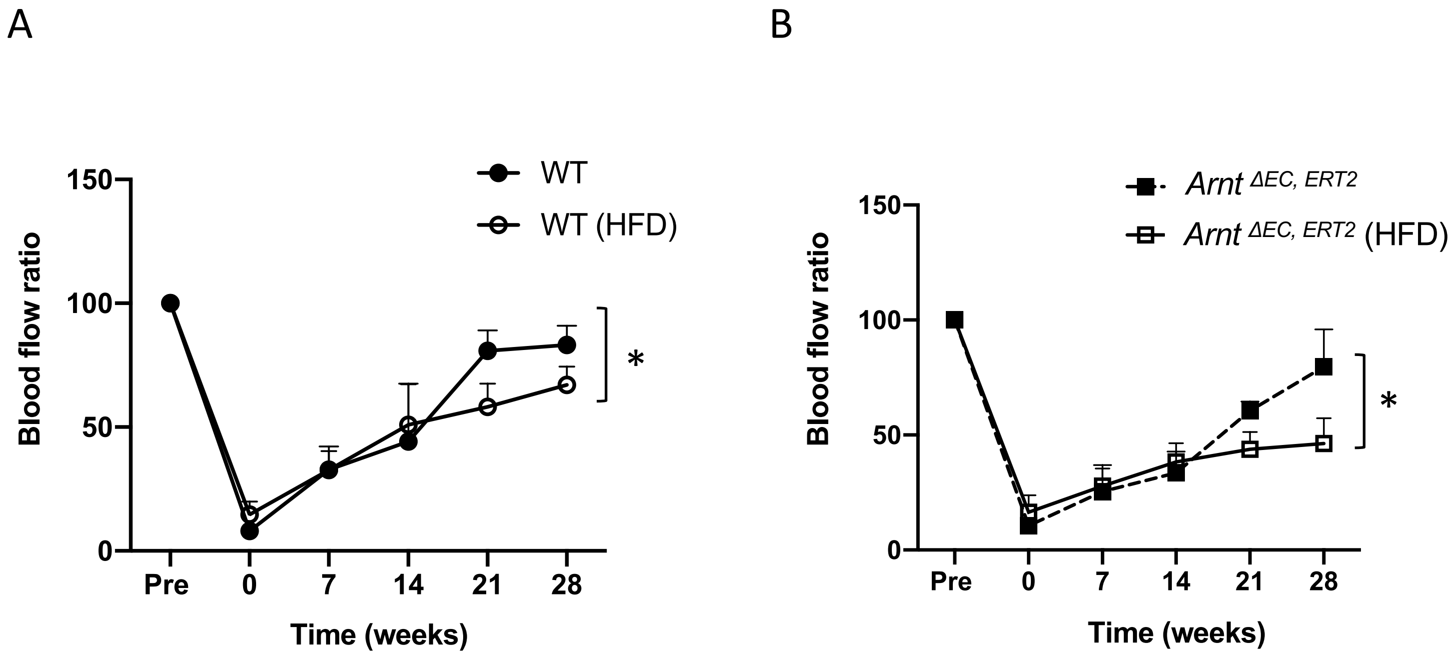

Supplemental figure 4. Quantitation of blood flow expressed as the ratio of blood flow in the ischemic (left) hindlimb to that in the standard (right) hindlimb. A) WT mice with and without 8 weeks of 60% high-fat diet (HFD). B) *Arnt* $^{\Delta EC, ERT2}$ mouse with and without 8 weeks 60% high-fat diet (HFD).Data are means of values from 8-11 animals per group. *P<0.01 versus corresponding control.

A

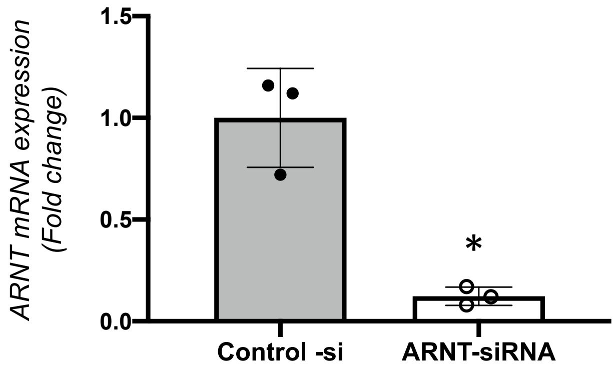

B

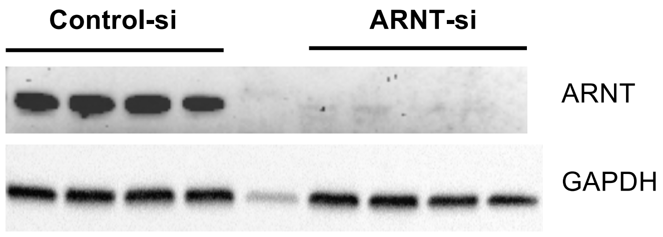

Supplemental figure 5. siRNA induced ARNT deletion in HUVECs. A) ARNT mRNA expression in HUVECs with either control-siRNA or ARNT-siRNA for 48 hours. n=3. *P<0.001. B) ARNT protein expression by Western blot (n=4). GAPDH served as an internal control.
